# The Influence of Synthesis Conditions on the Antioxidant Activity of Selenium Nanoparticles

**DOI:** 10.3390/molecules27082486

**Published:** 2022-04-12

**Authors:** Aleksandra Sentkowska, Krystyna Pyrzyńska

**Affiliations:** 1 Heavy Ion Laboratory, University of Warsaw, Pasteura 5A, 02-093 Warsaw, Poland; 2 Department of Chemistry, University of Warsaw, Pasteura 1, 02-093 Warsaw, Poland; kryspyrz@chem.uw.edu.pl

**Keywords:** selenium, selenium nanoparticles, antioxidant activities, clean-up procedure

## Abstract

Selenium nanoparticles (SeNPs) have attracted great attention in recent years due to their unique properties and potential bioactivities. While the production of SeNPs has been long reported, there is little news about the influence of reaction conditions and clean-up procedure on their physical properties (e.g., shape, size) as well as their antioxidant activity. This study takes up this issue. SeNPs were synthesized by two methods using cysteine and ascorbic acid as selenium reductants. The reactions were performed with and without the use of polyvinyl alcohol as a stabilizer. After the synthesis, SeNPs were cleaned using various procedures. The antioxidant properties of the obtained SeNPs were investigated using DPPH and hydroxyl radical scavenging assays. It was found that their antioxidant activity does not always depend only on the nanoparticles size but also on their homogeneity. Moreover, the size and morphology of selenium nanoparticles are controlled by the clean-up step.

## 1. Introduction

Selenium is an essential trace element that supports many processes that occur in human body. The importance of selenium in the human diet comes from the fact that it is involved in protection of the cell from oxidative damage and plays a key role in decreasing lipid peroxidation [1,2]. Moreover, selenium is postulated to be useful for protection against various forms of cancer and many serious diseases, including cardiovascular disease, arthritis and muscular dystrophy [3,4]. For that reason, selenium dietary supplements generate considerable interest in pharmaceutical and food sciences [5]. Some of the selenium supplements, particularly the inorganic forms, have shown toxicity in higher nutritional doses. However, it is necessary to emphasize that the biological activity of the selenium depends on its chemical form and structure. In recent years, selenium nanoparticles (SeNPs) have attracted great attention due to their unique properties and potential bioactivities. The toxicity of SeNPs is significantly lower in comparison to that of inorganic and organic forms of selenium [6]. SeNPs offer great potential for several applications in the fields of medicine, diagnostics, therapeutics and toxicology [7,8,9,10,11]. On the other hand, due to their unique structural, optical and electronic properties, selenium nanomaterials also find applications in electronics and technology. The newest research shows great potential of SeNPs regarding their antimicrobial activity. Various mechanisms of their action were described, including the generation of radical oxygen species (ROS), interaction with cell barrier or inhibition of the synthesis of proteins and DNA [12]. The ROS species (e.g., hydroxyl radicals, superoxide anions, hydrogen peroxide) can inhibit DNA replication or amino acid synthesis, but they can also damage bacterial cell membranes. The potential of nanoparticles (NPs) as antimicrobial agents can be explained by their ability to simultaneously act through these multiple mechanisms. In such a situation, microbes are unable to develop resistance to these expressed mechanisms of action, contrary to the case for commercially available antibiotics [12]. The particle size, shape and surface morphology are important parameters determining the interaction of nanomaterials with bioorganisms. Due to these findings, the synthesis method is crucial when the obtained SeNPs are intended for use in the biomedical field. 

Several chemical and physical methods have been proposed to prepare SeNPs. The main method of preparing nanoselenium is the chemical reduction of selenium salts [6,13,14,15,16]. Sodium selenite, sodium selenosulfate or selenious acid are examples of the precursors in the chemical synthesis of SeNPs. However, chemical methodologies are criticized due to the use of toxic chemicals in the synthesis protocol. Attempts have been made to use non-toxic reagents in the synthesis of SeNPs such as ascorbic acid [13,14,17,18] or sugars [13,18,19]; however, such actions are limited by the instability of the nanoparticles. This can be improved by adding stabilizers such as glucose, chitosan or polyvinyl alcohol (PVA) [13,14,20]. This is a very important aspect of nanoselenium synthesis because SeNPs are usually prone to agglomeration into large clusters in aqueous media, which results in a reduction in their bioactivity, biocompatibility and bioavailability [20]. On the other hand, some residuals of the used stabilizers limit the application of the obtained SeNPs in pharmaceutical and medicinal areas. Therefore, green synthesis methods of SeNPs preparation have recently garnered great attention [21,22,23,24,25,26]. These ecofriendly methods involve plant extracts or various microorganisms in their protocol. The main assumption of such methods is the use of naturally occurring substances in the extract as both stabilizers and reductants [13]. Many studies showed that SeNPs possesses higher antioxidant activity than the used plant extract itself [27,28]. It is known that particular synthesis conditions (e.g., concentration and type of selenium precursor and reductant, presence of stabilizer, temperature) affect the size, shape and stability of the obtained nanoparticles [6,14,29,30,31]. However, until now, it was not reported how the clean-up procedure of previously synthesized SeNPs affects their physical properties such as size and shape and, even more importantly, their antioxidant properties. Usually, the reaction protocols involve stirring of the reaction mixture for different time intervals and, in some cases, also heating at the desired temperature [30,31,32]. The most common way to purify and isolate NPs from surrounding liquid is centrifugation at different speeds and then washing with water [23,24,33,34,35]. However, the duration and speed of centrifugation (expressed as revolutions per minute (rpm)) are different in each study. In the research described by Sharma et al., SeNPs were centrifuged and precipitated at 15,000 rpm [34]. As a result, selenium nanoballs of a size of about 3–18 nm were obtained. Menon et al. centrifuged the reaction mixture at 10,000 rpm for 10 min and then washed three times with Mili-Q water [23]. As a result, shaped NPs in range 100–150 nm were obtained. SeNPs were also collected by centrifuging the solution at 12,000 rpm, and the pellet was resuspended in sterile double-distilled water before using in a bacterial experiment [35]. Centrifugation was also used by Chen et al. [36]. In this study, the post reaction solution was aged for 24 h followed by centrifugation at 9000 rpm for 30 min. Then, the precipitate was washed with twice with water and ethanol. At the end, the final product was redispersed in deionized water. In other studies, Chen et al. mentioned overnight dialysis against ultrapure water as a potential method for the purification of SeNPs [37,38]. This was a critical operation, due to the fact that, in the next stages of the research, the antioxidant properties of the SeNPs were tested. However, the impact of such a cleaning procedure on the physical properties of SeNPs was also not investigated. SeNPs have been considered as a potential anticancer and antioxidant agent. Before NPs can be used in treatment, they must undergo an entire clinical trial process similar to that of drugs [39]. One of the most challenging steps is the adaptation of the synthesis method from laboratory to industrial scale. The use of SeNPs in clinical trials depends on the approval of the production methods and quality assurance of the final product by the implementation and verification of good manufacturing practice (GMP). GMP is strictly connected with the quality assurance (QA) process, which should be implemented to guarantee that the obtained NPs follow the specifications and meet the required quality. Compliance with quality control (QC) requires SeNPs to be tested to confirm that they are of expected quality for the intended use. From the point of view of these regulations, it is obvious that the type and concentration of the used reductant must be characterized in detail, because it has enormous impact on the properties of the synthesized SeNPs. On the other hand, the very possibility of influencing the properties of SeNPs also at the purification stage gives enormous opportunities to improve some methods of SeNP synthesis so that they meet the requirements set by GMP and QC. Additionally, if they have such a large impact on the parameters of NPs, their optimization should be one of the points taken into account when developing GMP standards for SeNPs. It should be remembered that the methods of purifying NPs may not only improve their properties but also worsen them. Thus, there is the need to develop an optimal method for the purification of the synthesized NPs, as this step ultimately determines the quality of the obtained product. However, there is no comparison of the properties of NPs obtained with the same method but with different purification steps. 

In this study, SeNPs were synthesized using two well-known chemical methods, involving ascorbic acid (AA) or cysteine (Cys) as the reductants and sodium selenite as the precursor. The syntheses were carried out in two variants: in the presence and without the use of polyvinyl alcohol (PVA) as a stabilizer. After each synthesis, SeNPs were cleaned by rinsing with water and centrifuging at different speeds (8000 and 12,000 rpm). The impact of additional post-synthesis heating on their physical properties was also investigated. The influence of all the above-mentioned factors on the antioxidant capacity of SeNPs has been evaluated. To the best of our knowledge, this has not been described so far. This step of SeNP synthesis is extremely important if the obtained SeNPs are intended for use in medicine, e.g., as a substitute for antibiotics [40]. 

The antioxidant properties of the synthesized SeNPs were examined using scavenging of the 2,2-diphenyl-1-picrylhydrazyl (DPPH) as well as hydroxyl (OH) radicals. The morphology of the SeNPs was compared using scanning electron microscopy (SEM) and transmission electron microscopy (TEM). 

## 2. Results and Discussion

**Figure 1:**
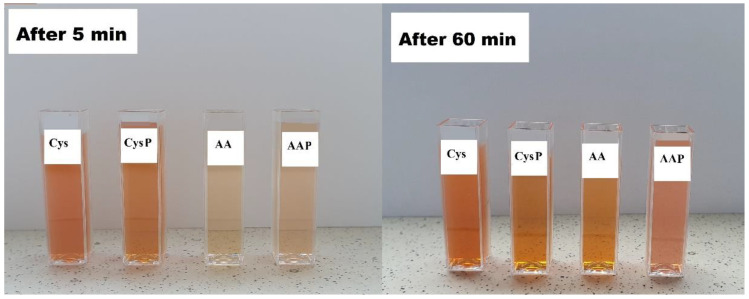
Color changes of product solutions upon synthesis of SeNPs (CysP or AAP—reductant in the presence of PVA).

Syntheses of SeNPs were carried out in parallel by the reduction of sodium selenite using AA or Cys. Theoretically, the antioxidant properties of SeNPs can already be controlled at the stage of selecting the concentrations of the reactants for their synthesis. Li et al. [41] found that an excess of Cys to Se(IV) that was higher than 1:4 caused the aggregation of SeNPs. Such a process would be disadvantageous from the point of view of the antioxidant capacity of SeNPs. It has been proven that the antioxidant capacity of NPs increases with the decrease in their dimensions [42]. Therefore, a selenium to reductants ratio of 1:4 was selected for use. After mixing the reagents, the color of the product solutions gradually changed from colorless through light orange, to orange and brick red due to reduction of selenium ions, which confirmed SeNP formation (Figure 1).

**Figure 2:**
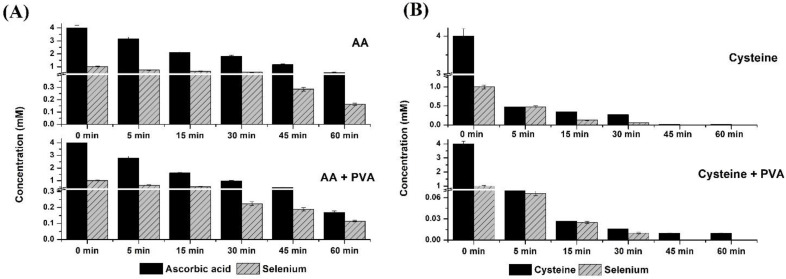
The changes in the concentration of selenium salt and reductants: (**A**) AA and (**B**) Cys as a function of synthesis time.

**Figure 3:**
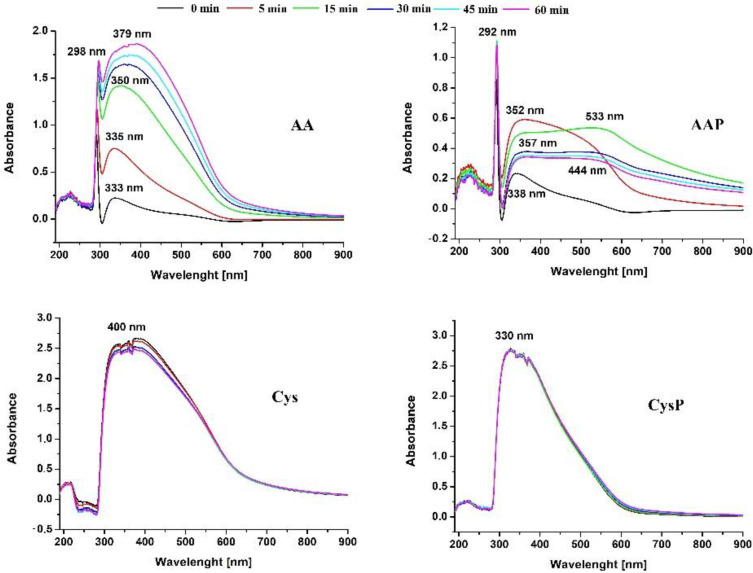
UV–visible spectra of SeNPs as a function of synthesis time.

The synthesis of SeNPs was also monitored by determining the decrease of the reagent’s concentration in the supernatant using hydrophilic interaction chromatography coupled with mass spectrometry detection. For each compound used in the synthesis, the optimum conditions of MRM (multiple reaction mode) were determined. The obtained MS spectra are presented in Figure S1. Figure 2 shows the remaining concentration of selenium ions and AA or Cys at various time intervals during synthesis. The appropriate chromatograms as well as mass spectra of the reagents are shown in Figure S1. The obtained results a confirmed faster reaction rate with Cys. Its concentration decreased from an initial value of 4 to 0.07 mg L^−1^ after 5 min of reaction time; thus, the reduction of Se(IV) by Cys is almost instantaneous. In the presence of PVA as a stabilizer, this reaction is slightly slower. Cys was found in the reaction media at the level of 0.48 mg L^−1^ after 5 min of mixing with 88% synthesis yield. After 30 min of reaction with AA, the synthesis yields of SeNPs were found to be 68% and 77% in the absence and presence of PVA, respectively. Even after 60 min of intensive mixing with a stirring speed of 1000 rpm, there was still a small residue of both components in the reaction medium. The higher intensity of the transformation process using Cys was also confirmed on the basis of changes in the UV–Vis absorption spectra during the ongoing synthesis (Figure 3). 

Lin et al. [43] postulated that the well-known belief that nano-Se is “red” is somewhat misleading. In fact, the color of the NPs’ suspension strictly depends on their dimensions. These observations were the basis for the authors to develop a method for determining the size of SeNPs based on their UV–Vis spectra [43]. According to this method, the suspension of the SeNPs of 20 nm diameter had a yellowish-orange appearance and showed the absorption maximum below 250 nm. When the particle sizes increase, the characteristic red-shift of the absorbance peak maxima is observed. As result, SeNPs’ suspension with diameters of about 100 nm was characterized by an absorption maximum below 350 nm, while for SeNPs’ with diameters of 240 ± 32.2 nm, the absorbance maximum was observed at 680 nm. This method was also used in our study. 

The obtained results indicate that the extension of the reaction time does not cause the changes in the spectra obtained for solutions of Cys as a reductant (Figure 3). This suggests that SeNPs obtained by this method do not change their dimensions with increasing mixing time. Therefore, they probably do not aggregate. Any change in the size of the obtained NPs would entail a change in the spectrum, which was not observed here. In the case of synthesis with Cys and CysP, the UV–Vis spectrum did not change within two hours from the end of the synthesis, and the stability of SeNPs was observed during this time interval. Based on the Lin et al. method [43], the predicted diameters of obtained Cys SeNPs were about 101 ± 9.8 nm. However, the shift of the maximum absorbance peak for the CysP suggests that SeNPs obtained this way are slightly smaller. This is in good accordance with our studies, in which size ranges equal to 108 ± 9.3 nm for Cys and 80 ± 5.1 nm for CysP were obtained. A totally different observation was made for the synthesis with the use of AA. In this case, changes in the location of the maximum absorbance peak were observed during the extension of the reaction time. This may be due to the slower reaction rate, which was confirmed by monitoring the decrease in the concentration of the reactants (Figure 2). As explained later in the publication, the process of NPs synthesis is a dynamic one, and depending on which mechanism prevails, changes in the size and structure of the NPs are observed. These changes are visible in the UV–Vis spectrum. For the synthesis with AA, the increasing absorbance of the peak located at 400 nm was observed, which suggests that larger of SeNPs are expected. Our research has proven the formation of SeNPs with the size of 193 ± 9.5 nm. The presence of a stabilizing agent greatly differentiates the sizes of NPs. The peak at 300 nm indicates the presence of particles of the size 70 ± 9.1 nm according to the Lin model and 90 ± 7.3 nm based on our studies. However, much bigger SeNPs can also be found—predicted to be 182.8 ± 33.2 nm and 189 ± 6.4 nm based on our study. It can be concluded that the use of a stabilizer does not always bring the desired effect. In the reaction with Cys, slightly lower dimensions of NPs were obtained when the PVA was used. However, in the synthesis with AA, the presence of PVA made the NPs less homogenous, with a predominance of larger particles. 

It is worth noticing that the occurrence of the SeNPs’ absorption spectra with maxima within visible and UV regions was often explained by the formation of surface plasma vibration on spherical NPs [13,22,24,33,44,45]. Probably, the authors of these works just copied it from reports on noble-metal NPs. Selenium, as a semiconductor, lacks free conduction electrons. The light irradiation of SeNPs can cause exciton resonance or transition to occur [46], determining the development of their unique optical properties.

In the past decade, prompted by rapid developments in nanotechnology, SeNPs have attracted extensive attention from researchers in biomedical fields [23,40]. SeNPs obtained by chemical synthesis should be cleaned first to be suitable for use in these applications, as residues of the reagents may be toxic or can block the desired effect of the nanomaterial. However, systematic studies on the impact of cleaning procedure on the properties of SeNPs are very rare. For evaluation of the antioxidant activity of SeNPs, an appropriate volume of the formed colloidal solution was just mixed with a given reagent [14,23,47]. In such a case, the presence of the used reductant residue could affect the results. 

**Figure 4:**
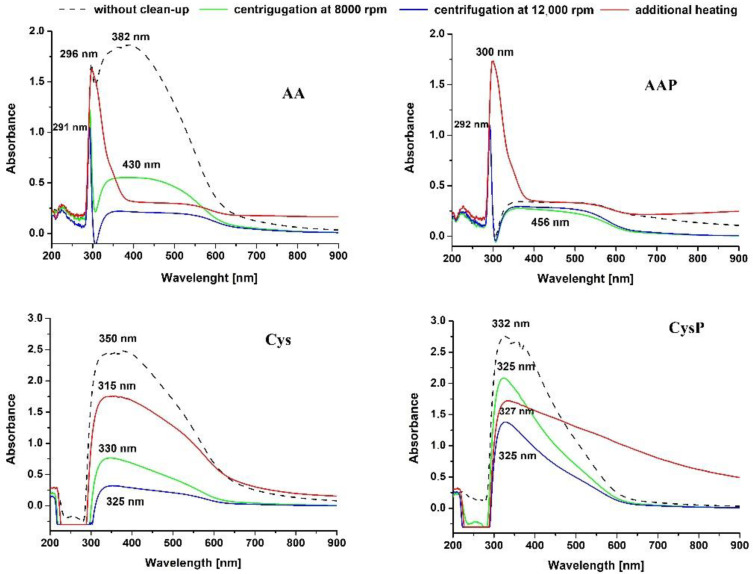
UV–visible spectra of SeNPs after the application of various cleaning procedures.

In our study, two centrifugation speeds 8000 and 12,000 rpm were used. The influence of the cleaning procedure on the properties of SeNPs can already be observed in the UV–Vis spectra of cleaned NPs (Figure 4). A wide band in the range of 350–550 nm was observed for AA SeNPs, which corresponds to the NPs in the size range 195 ± 5.0 nm. In the case of the AAP, the band at 382 nm exhibited lower intensity with a simultaneous increase in the intensity of the band at about 300 nm. This suggests that PVA prevents agglomeration of the resulting SeNPs, hence shifting the absorption maxima toward lower wavelengths. Moreover, the use of any purification method in the case of AA SeNPs led to a reduction in their size, as evidenced by the disappearance of the absorption band at 382 nm. It should be mentioned that other researchers have also reported a similar type of SeNP multimodal distribution [15,47,48]. Heating at different temperatures (70–120 °C) was also employed during the synthesis of SeNPs [32,48,49]. Zhang et al. found that heating treatment (1 h at 90 °C) caused aggregation of SeNPs into larger sizes and rods, which leads to a significant reduction of their bioactivity in mice [50]. In two synthesis methods using AA, heating of the post-reaction mixture led to an increase in peak intensity with an absorption maximum of 300 nm. This is a desirable effect, as this increases the formation intensity of NPs with dimensions of about 100 nm. 

For synthesis conducted using Cys, the occurrence of a peak at 325–350 nm indicates the formation of SeNPs with lower diameters (108 ± 4.2 nm). The highest intensity was observed for NPs without a clean-up procedure, while the lowest observed for those centrifuged at high speed (12,000 rpm, 17,257 rcf), while tailoring the signal to higher wavelengths. This suggests that centrifugation does not prevent the aggregation of NPs but makes them less homogeneous with a predominance of NPs larger than 100 nm. It should be highlighted that heating the post-reaction mixture resulted in a reduction in the signal intensity of about 300 nm, and additionally, a significant tailing of the signal was observed, if PVA was used for synthesis. Surprisingly, none of the purification methods of SeNPs obtained from Cys synthesis seemed to improve their properties but rather favored their agglomeration. 

**Figure 5:**
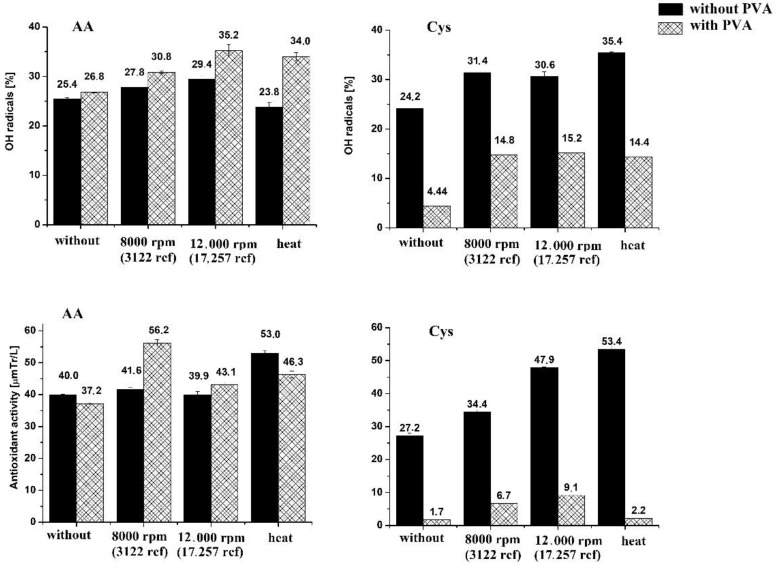
The antioxidant activity of the obtained SeNPs.

According to previous reports, SeNPs are strong free radical scavengers that can be used as an active form of selenium in food supplements [14,23,24,45,47,51]. As mentioned before, the antioxidant capacity of NPs increases with the decrease in their dimensions [42]. This leads to the hypothesis that, since the cleaning procedure affects the size of the NPs, it also has an impact on their antioxidant capacity. However, the impact of these procedures on the antioxidant properties of SeNPs has not been reported on. Thus, we compared the free radical scavenging potential of SeNPs toward hydroxyl and DPPH radicals. Stable DPPH radicals are widely used to evaluate the antioxidant activity of NPs [52]. The scavenging of hydroxyl radicals is an important antioxidant activity because of the very high reactivity of the OH radical, enabling it to react with a wide range of molecules found in living cells. The applied cleaning procedure was not changed and involved centrifugation at two speeds (8000 and 12,000 rpm) or additional heating (1 h at 70 °C). The obtained results are presented in Figure 5.

At the beginning, the antioxidant activity of the used reagents was established. In the DPPH assay, the antioxidant activity of AA (in the same concentration as in SeNP synthesis) was 1.15 ± 0.11 µmol TR L^−1^. The addition of PVA did not change this value; in its presence, the value 1.16 ± 0.10 µmol TR L^−1^ was obtained. The same situation was observed for Cys, for which, the determined value of the antioxidant capacity was 1.11 ± 0.05 µmol TR L^−1^. PVA itself shows a very low antioxidant capacity of 0.008 ± 0.0001 µmol TR L^−1^. In the OH radical assay, the addition of PVA resulted in an increase in capacity, from 93.0% to 100% in case of Cys solution and from 33.3% to 98.1% for AA. This suggests a strong synergistic effect between the reactants. However, CysP SeNPs showed dramatically lower antioxidant capacity than those obtained from analogous synthesis but without the use of a stabilizer. Under these conditions, differences were obtained at a significant level (*p* = 0.05). Such differences were not observed in the case of synthesis with AA as a reductant. The use of any method of purification of NPs obtained with the use of AA did not significantly affect the antioxidant capacity they demonstrated. Only the heating of the reaction mixture increased these capabilities, but in this case, a higher effect was observed for the AA SeNPs. In the case of Cys SeNPs, applying any cleaning procedure increased their ability to scavenge DPPH radicals. As previously observed for the SeNPs from the synthesis with AA, the highest increase was observed for the synthesis protocol that involved heating. Such observations can suggest that the greater impact on the SeNPs’ antioxidant activity was due to their homogeneity and not their size. If their dimensions were crucial, a significant increase in antioxidant capacity should have been observed when using high-speed centrifugation (12,000 rpm). Such a procedure prevents the aggregation of SeNPs, while heating makes them more spherical and homogeneous. The lowest results in the DPPH assay were obtained for CysP (1.7–9.1 µmol TR L^−1^), which further confirmed our predictions as the stabilizer also prevents the aggregations of SeNPs, but the small dimensions of NPs are not the dominant factor affecting their antioxidant capacity.

The influence of the cleaning procedure for SeNPs on the scavenging of hydroxyl radicals was also positive. The highest ability to scavenge OH radicals was established for AAP SeNPs cleaned using high-speed centrifugation (35.2% ± 0.9%). This value was not statistically different from that obtained for Cys subjected to additional heating (35.4% ± 0.5%). Presence of the stabilizer during the synthesis of SeNPs with Cys, resulted in lower ability to scavenge the hydroxyl radicals; however, every cleaning procedure, applied just after the synthesis resulted in increased antioxidant capacity. For the synthesis of SeNPs with AA, the presence of a stabilizer resulted in a higher ability to scavenge OH radicals. Every clean-up procedure improved this ability, with the exception of additional heating of the post-reaction mixture without the stabilizer.

**Figure 6:**
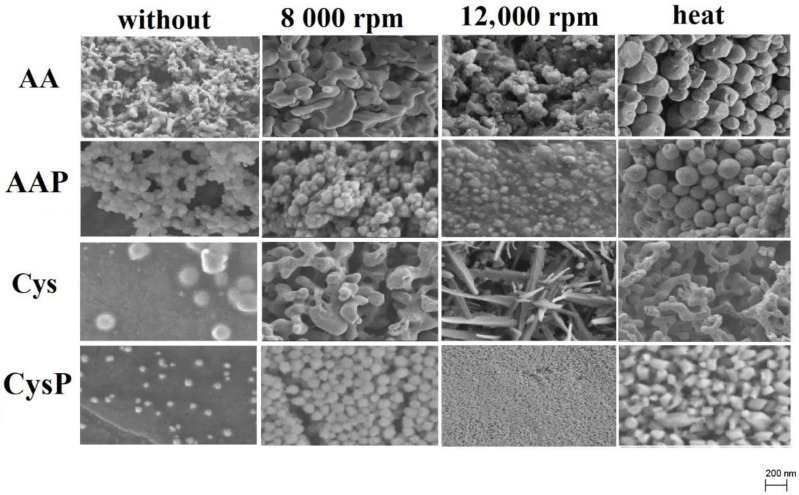
SEM images of the obtained SeNPs.

The morphology of synthesized SeNPs was evaluated by SEM analysis, and majority of them were found to be spherical (Figure 6). Only the Cys SeNPs centrifuged at high speed were nanorods. Similar shape of SeNPs was obtained with asparagines and polyvinylpyrrolidone [35]. Surprisingly, the highest antioxidant abilities were not determined for the most spherical SeNPs obtained for Cys. The highest ability to scavenge free radicals was found in SeNPs obtained without the use of PVA but subjected to additional heating; however, their average size increased at elevated temperature. This was caused by the aggregation of the NPs. A similar phenomenon was observed by others [31,48]. Literature reports indicate that smaller SeNPs exhibit higher antioxidant activity [34,50], but this correlation was observed only for AAP. Centrifugation reduced only reduced the size of AAP SeNPs, and these showed the highest ability to scavenge free radicals of all the protocols examined for synthesis involving AA. On the other hand, Zhang et al. demonstrated that SeNPs with different sizes (5–200 nm) have equal capacity in the induction of selenoenzymes in cultured cells and in mice [49]. Our results, rather, indicate that SeNPs with better homogeneity show higher scavenging activity toward free radicals. It should be recalled that synthesis of SeNPs is a dynamic process, which is controlled by both thermodynamics and kinetics. Thermodynamics deals with the driving force of a system moving from the initial state to the product state, whereas kinetics is concerned with the energy barriers of the specific pathways in this process [53]. Due to this fact, SeNPs are changing during synthesis to reach a relatively stable state. This can be explained by a well-known concept called Ostwald ripening. When the substrates used for synthesis are nearly depleted, some of the NPs can redissolve in the reaction solution. Such dissolved components can attach to the surface of the NPs, increasing their size. As a result, fewer but larger NPs are formed. Another phenomenon that can also take place during NPs synthesis is digestive ripening. In this process, larger NPs are transformed into smaller ones with a uniform monodisperse state by mixing NPs in a solution that contains digestive ripening ligands, e.g., PVA. The action of these two mechanisms is visible in the surface changes of NPs, as shown in Figure 6. When the results obtained for Cys are discussed, the larger SeNPs are broken up into smaller ones, which was induced by PVA. Then, these CysP were completely transformed into a nearly monodisperse form with an almost uniform size by centrifuging at 12,000 rpm. When centrifuging at 8000 rpm, monodisperse particles together with larger particles are formed. Thus, it can be inferred that digestive ripening is responsible for such an observation. On the other hand, probably due to the weak binding of PVA with SeNPs’ surfaces, uniform SeNPs are transformed back to larger polyhedral particles as a result of heating, according to the mechanism of Ostwald ripening. Such a dynamic state is also observed for the SeNPs obtained via synthesis with AA. Using this phenomenon, the production of nanorods in the case of Cys centrifuged at 12,000 rpm can also be explained. Such nanostructures are synthesized in solution at low temperature mainly due to the wet-chemical reactions. The orientation and shape of nanorods can be controlled mostly by temperature, concentration of precursor and the length of the rod by the time of synthesis. In our case, only the method of cleaning previously formed SeNPs was changed. It can be assumed that centrifuging at high speed favors the process of Ostwald ripening. However, the mechanism in this case is different than for other cleaning procedures due to a combination of ligands that act as the shape-control agents and bond to different facets of the nanorod with different strengths. This allows different faces of the nanorod to grow at different rates, producing an elongated object.

**Figure 7:**
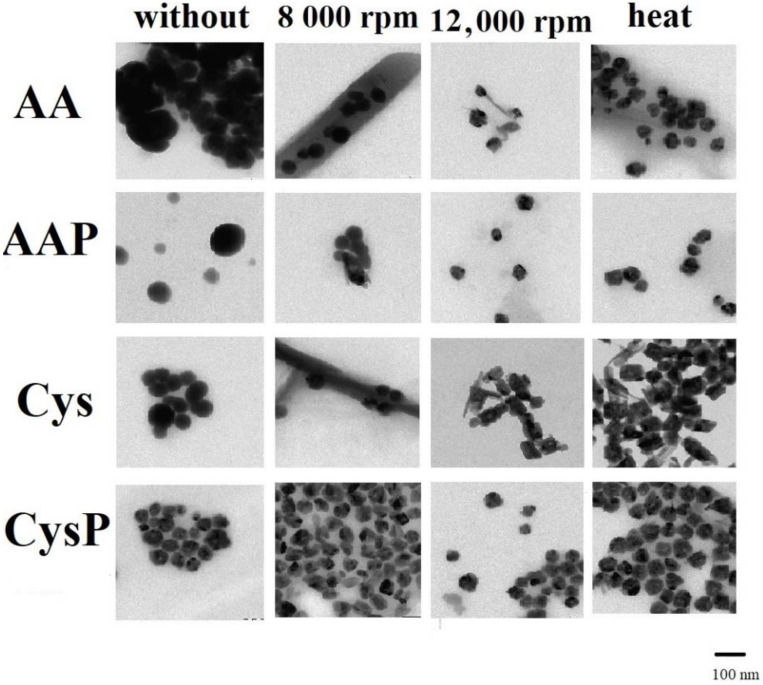
TEM images of the obtained SeNPs.

Figure 7 shows the TEM images of the obtained SeNPs. These results confirm that more spherical-shaped NPs were obtained without any purification procedure. Chen et al. found that the reaction temperature affects not only the size evolution of SeNPs but also their shape formation [48]. The TEM images were used for the construction of histograms of SeNP size distribution in each obtained sample. The results are presented in Figure S2. The obtained results confirmed the enormous influence of the cleaning procedure on the size of the NPs. In many cases, the applied procedure resulted in obtaining less-homogeneous particles of various sizes. This is especially evident when additional heating is used for AA and CysP. We previously linked the antioxidant capacity with the homogeneity of NPs. Referring to these results, this influence is particularly visible. A lower hydroxyl scavenging capacity was observed for the sample of AA subjected to heating than for the sample without the cleaning procedure. The use of heating also resulted in the reduction by half of the ability to neutralize hydroxyl radicals for CysP. On the other hand, it was observed that, for some samples with less homogeneity but with a predominance of smaller NPs, the antioxidant capacity was higher in comparison to that of the more homogeneous samples. Such a situation was observed for AA subjected to moderate-speed centrifugation (8000 rpm) or heating. This suggests that the antioxidant capacity of SeNPs is influenced by both size and homogeneity. Both these factors are important. Such relations can be also observed for the DPPH radical scavenging. For example, higher antioxidant activity was observed for the more homogenous sample of AAP, subjected to 8000 rpm, than for the sample without any cleaning procedure but with the predominance of smaller particles. 

## 3. Materials and Methods

### 3.1. Reagents

The commercial standards of sodium selenite (Na_2_SeO_3_) as well as ascorbic acid (AA), L-cysteine (Cys) and polyvinyl alcohol (PVA) were purchased from Merck-Sigma (Steinheim, Germany). Ultrapure water from Milli-Q system (Millipore, Bedford, MA, USA) was used in all experiments.

### 3.2. Synthesis of Selenium Nanoparticles and Their Purification

SeNPs were synthesized via reduction of Na_2_SeO_3_ with ascorbic acid. The procedure was performed with and without polyvinyl alcohol (PVA) as a stabilizing agent. PVA is often used for this purpose [32]. Briefly, 20 mL of sodium selenite solution (5 × 10^−3^ mol L^−1^) was placed in a beaker with a magnetic stirrer. Then, 10 mL of AA solution (40 × 10^−3^ mol L^−1^) was added dropwise. After 60 min of stirring, 70 mL of Milli-Q water was added. In the second procedure of synthesis, PVA was added just after the first addition of AA in such amount that its final concentration was 1 mg L^−1^. 

SeNPs were also synthesized using Cys as the reductant. In detail, to 0.1 mol L^−1^ solution of Na_2_SeO_3_, Cys solution (50 × 10^−3^ mol L^−1^) was added dropwise with vigorous stirring (stirring speed of 1000 rpm) for 60 min. In parallel, SeNPs were obtained using PVA as a stabilizing agent. In each method of synthesis, the final concentration of selenium was equal to 1 × 10^−3^ mol L^−1^, and the reductants was equal to 4 × 10^−3^ mol L^−1^. 

After the synthesis, SeNPs were purified from the surrounding liquid containing dissolved substrate residuals using different procedures: (i) centrifugation for 10 min with two units of rotational speed 8000 rpm (3122 rcf) and 12,000 rpm (17,257 rcf), decantation and rising three times with 10 mL of deionized water; (ii) additional heating with magnetic stirring at 70 °C for 1 h, then rising three times with 10 mL of deionized water. Heating the reaction mixture is sometimes recommended in order to obtain more-homogeneous NPs [40]. The morphology and properties of SeNPs synthesized using these clean-up procedures were compared with those of SeNPs obtained directly by mixing reagents. 

### 3.3. Characterization of Selenium Nanoparticles

The morphology and characterization of SeNPs were investigated using various methods. The size and shape of SeNPs were observed using transmission electron microscopy (TEM) with a TALOS F200 model (Thermo Fisher Scientific, Waltham, MA, USA) working at an accelerating voltage of 200 kV. A drop of bright red solution containing synthesized SeNPs was placed on copper grid and then air dried before examination. Scanning electron microscopy (SEM) was performed with a field-emission SEM (Merlin Zeiss) for images and morphology of SeNPs. Before measurements samples were plasma sputtered with a few-nanometer-thick Au/Pd layer. The obtained results were processed in the program iTEM, which is a part of the apparatus software. The histograms presented in the supplementary section were obtained based on the TEM images in the ImageJ program. 

The UV–visible absorption spectra were recorded in the range of 250–900 nm using a Perkin Elmer model Lambda 20 spectrophotometer with cuvettes of 1 cm length.

Chromatographic analysis was performed to study the conversion of reagents at various time intervals by determining their remaining concentration in the supernatant. It was done with the Shimadzu LC system consisting of binary pumps LC20-AD, degasser DGU-20A5, column oven CTO-20AC, autosampler SIL-20AC and 8030 triple quadrupole mass spectrometer (Shimadzu, Japan) equipped with an ESI source operated in negative-ion mode for selenium or in positive mode for ascorbic acid and cysteine quantification. The ESI conditions were as follows: capillary voltage 4.5 kV, temperature 400 °C, source gas flow 3 L min^−1^ and drying gas flow 10 L min^−1^. The separation was performed using a silica column Atlantis HILIC (100 × 2.1, 3 µm) from Phenomenex. The mobile phase consisted of methanol and water (85/15, *v*/*v*).

### 3.4. Antioxidant Activity

The hydroxyl radical scavenging activity of SeNPs was evaluated according to the method reported by Smirnoff and Cumbes [54]. The method involves the addition of 1 mL of SeNP solution to the reaction mixture containing: 1 mL iron sulfate (1.5 × 10^−3^ mol L^−1^), 0.7 mL hydrogen peroxide (6 × 10^−3^ mol L^−1^) and 0.3 mL sodium salicylate (2 × 10^−2^ mol L^−1^). After 60 min of incubation at 37 °C, the absorbance was measured at 562 nm, and the percentage of hydroxyl radical scavenging inhibition was calculated.

The radical scavenging ability was also examined in vitro using DPPH radicals [36]. Briefly, 0.1 mL of a sample was added to 2.4 mL of DPPH solution (9 × 10^−5^ mol L^−1^ in methanol). After 30 min, the decrease in the absorbance was measured at 518 nm. Each sample was analyzed in triplicate, and the results are expressed as Trolox equivalent (TRE) in µM.

## 4. Conclusions

The potential of nanopharmaceuticals, such as the investigated SeNPs, is based on their morphology, size and shape, and those parameters are related to the applied purification method. Our results clearly showed that every procedure that is used on the purification step has a great impact on the properties of the final product. In fact, NPs synthesized by the same method, under the same conditions, by the same person but subjected to different cleaning methods will have different physical properties. This, in turn, has an impact on their properties, including the antioxidant properties. It can be assumed that depending on the applied cleaning method, the properties that can be used in medicine are also subject to major changes. Therefore, it should be also optimized as a separate step on the way to obtain SeNPs with desired shape and properties. Such an approach can maximize the potential of SeNPs in medicinal applications. Moreover, when the intention is to evaluate the antioxidant activity of the obtained SeNPs, the clean-up procedure is crucial. The results described in the paper are a good introduction to biomedical research. It is assumed that they will be continued in this direction.

## Data Availability

The data presented in this study are available on request from the corresponding author.

## Supplementary Materials

The following supporting information can be downloaded at https://www.mdpi.com/article/10.3390/molecules27082486/s1, Figure S1: The chromatograms of Se(IV) and ascorbic acid or cysteine in the function of synthesis time.; Figure S2: Particle size distribution histograms of selenium nanoparticles.
