# Supplementary material for: The Influence of Synthesis Conditions on the Antioxidant Activity of Selenium Nanoparticles
Source: Molecules. 2022 Apr 12;27(8):2486. doi: 10.3390/molecules27082486 (PMC9026813; doi:10.3390/molecules27082486)
Supplement: Supplementary file 1 [file molecules-27-02486-s001.zip › molecules-1642400-supplementary.pdf]

molecules

Supplementary Material

The Influence of Synthesis Conditions on the Antioxidant Activity of Selenium Nanoparticles

Aleksandra Sentkowska and Krystyna Pyrzyńska

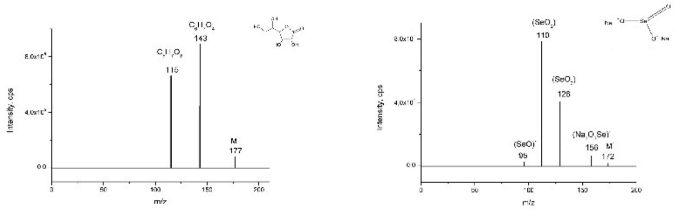

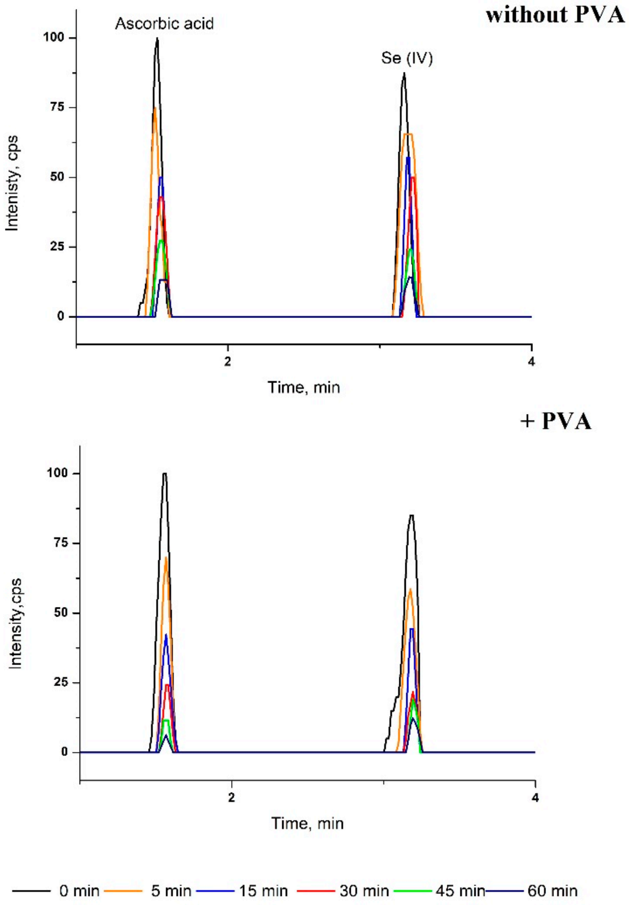

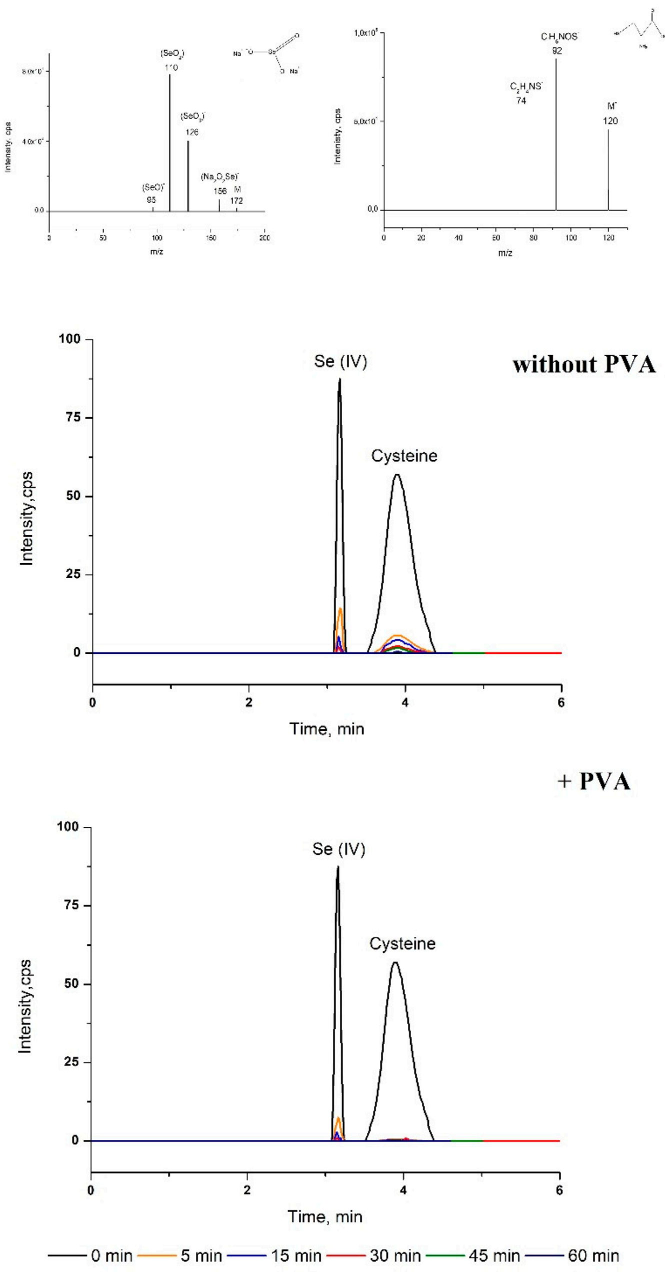

Figure S1. The chromatograms of Se(IV) and ascorbic acid or cysteine in the function of synthesis time.

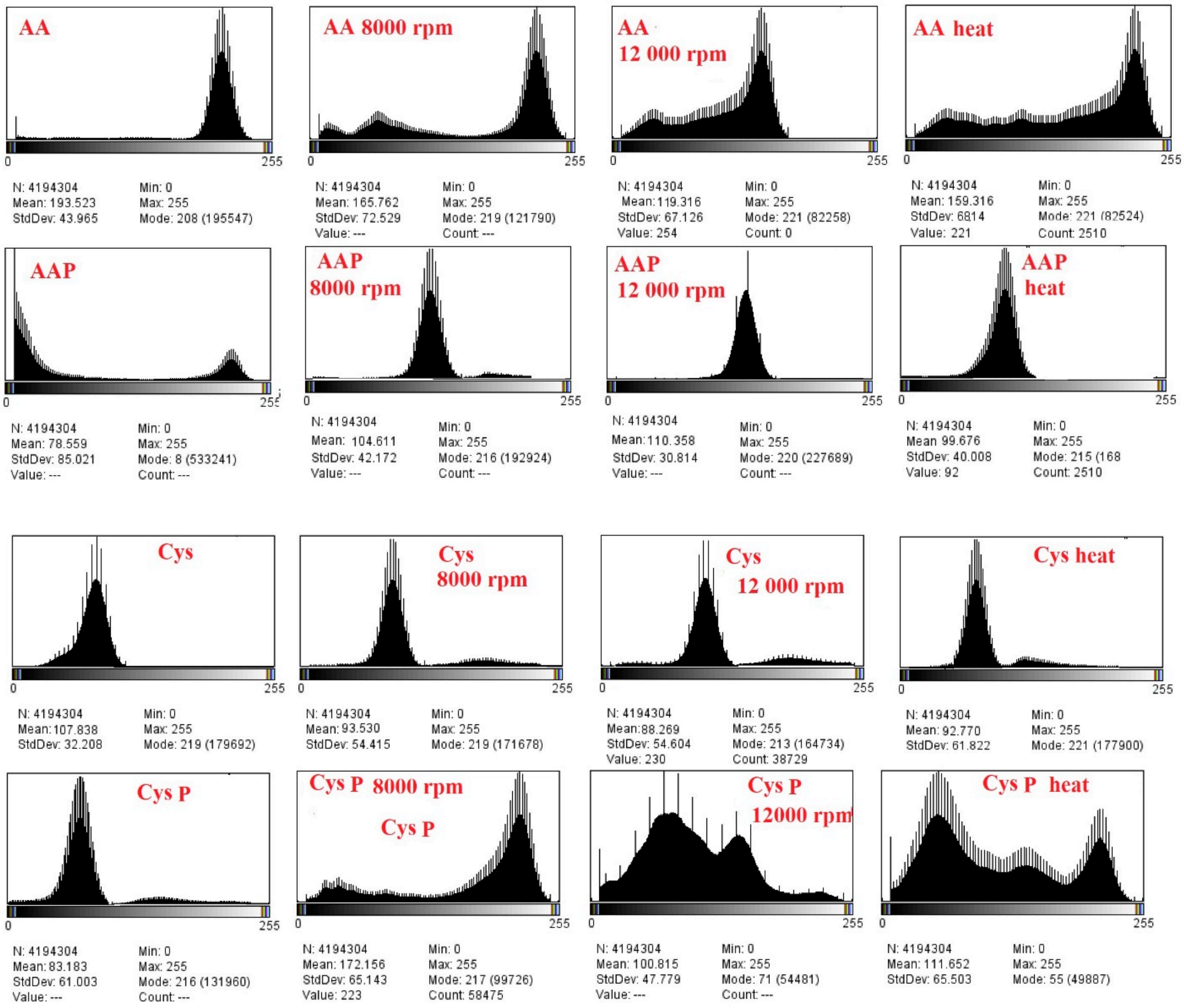

Figure S2. Particle size distribution histograms of selenium nanoparticles.
